# Specific organelle targeting as a key step in epidermal lipid synthesis

**DOI:** 10.3389/fmed.2026.1751515

**Published:** 2026-03-25

**Authors:** Margarita Schratter, Franz P. W. Radner

**Affiliations:** 1 Institute of Molecular Biosciences, University of Graz, Graz, Austria; 2 BioTechMed-Graz, Graz, Austria; 3 Field of Excellence BioHealth, University of Graz, Graz, Austria

**Keywords:** ABHD5, epidermis, ichthyosis, lipid droplet, omega-*O*-acylceramides, PNPLA1, protein localization, skin barrier

## Introduction

The epidermal barrier is among the most specialized lipid structures in human biology. Its integrity relies on the tightly coordinated synthesis of omega-*O*-acylceramides (acylCers) during keratinocyte differentiation (1–3). These complex lipids form a structural scaffold for both the corneocyte envelope and the extracellular lipid lamellae, which together maintain the barrier function of the *stratum corneum* (3, 4). Over the past decade, the enzymatic network responsible for acylCer biosynthesis has become increasingly well-defined. Central to this pathway are patatin-like phospholipase domain-containing 1 (PNPLA1), which catalyzes the final transacylation step (5, 6), and the lipid droplet (LD)-associated protein alpha/beta-hydrolase domain-containing 5 (ABHD5), which functions as its upstream regulatory cofactor (7, 8).

Recent work suggests that ABHD5 has a central function in epidermal lipid metabolism extending beyond the enzymatic activation of PNPLA1. Current evidence indicate that ABHD5 brings PNPLA1 into close proximity with triacylglycerol substrates in LDs, thereby enabling efficient acylCer synthesis (9). This model challenges the long-standing view of LDs as inert lipid reservoirs and instead defines them as dynamic metabolic platforms integrating epidermal lipid storage, mobilization, and barrier lipid synthesis.

In this Opinion, we discuss how these findings refine our current understanding of epidermal lipid biology. Specifically, we propose that the spatial organization of lipid-metabolizing enzymes, and not just their catalytic activity, can be an essential factor in maintaining epidermal homeostasis. From this standpoint, ichthyosis resulting from *ABHD5* mutations does not simply represent a metabolic defect, but rather an “organelle-targeting disorder,” in which the disruption of subcellular localization, rather than the loss of protein function, underlies disease pathogenesis, thereby broadening the mechanistic spectrum of inherited disorders of keratinization.

## From lipid storage to barrier synthesis

For many years, LDs have been considered as inert depots that simply store neutral lipids such as triacylglycerols and cholesteryl esters. However, this view has shifted fundamentally with the finding that LDs are dynamic organelles actively coordinating lipid metabolism and inter-organelle communication (10). In keratinocytes, this new concept has particular relevance, as epidermal LDs directly participate in the biosynthesis of acylCers, the lipids essential for barrier integrity (8, 9).

**Figure 1:**
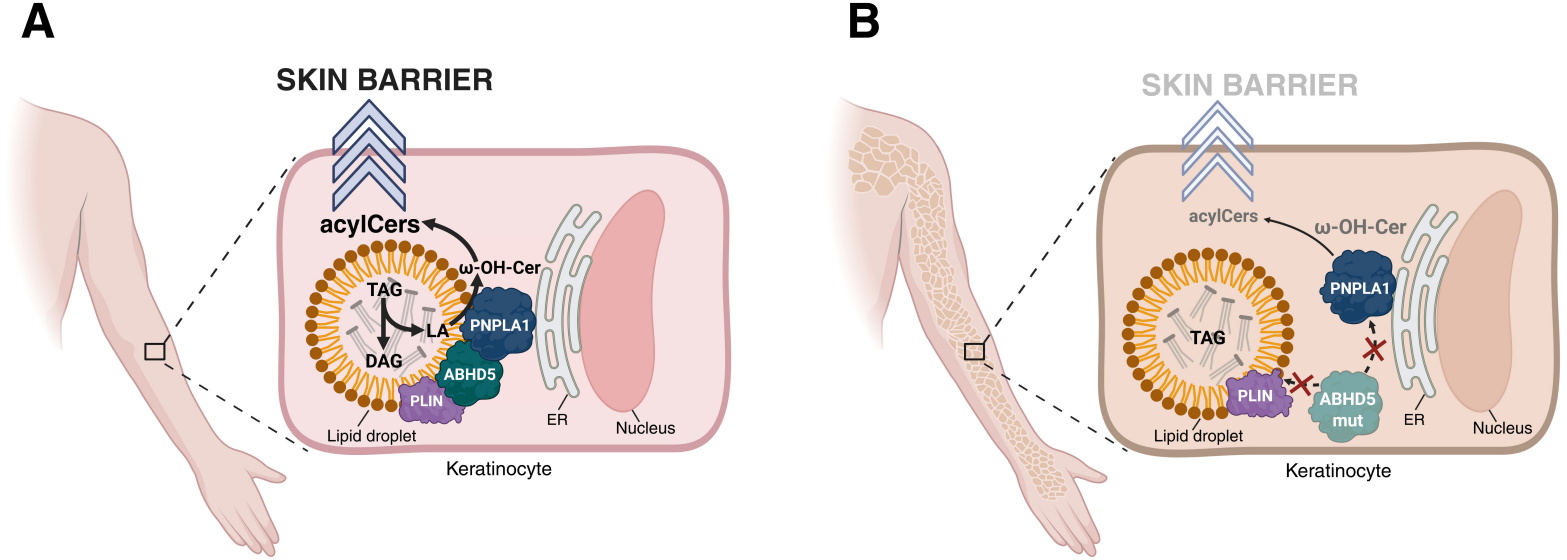
Proposed spatial model of ABHD5-PNPLA1 coordination in epidermal omega-*O*-acylceramide biosynthesis and its disruption in ABHD5 deficiency, based on recent experimental findings. **(A)** In healthy keratinocytes, ABHD5 functions as a spatial organizer that aligns PNPLA1 with the appropriate lipid environment for omega-*O*-acylceramide (acylCer) synthesis. PNPLA1 localizes mainly to the endoplasmic reticulum (ER) membrane, while ABHD5 associates with the hydrophobic surface of lipid droplets (LDs), likely via perilipin (PLIN)-mediated binding. In parallel, ABHD5 directly interacts with PNPLA1, thereby bringing LDs into functional proximity with the enzyme. This arrangement enhances the access of PNPLA1 to triacylglycerol (TAG)-derived linoleic acid (LA), which serves as the acyl donor for esterification of omega-hydroxy-ceramides (ω-OH-Cer) to generate acylCers, a process essential for epidermal barrier integrity. **(B)** Pathogenic variants of ABHD5 disrupt either the association of ABHD5 with perilipins and LDs or its interaction with PNPLA1. As a result, LDs are no longer appropriately aligned with PNPLA1, which limits substrate availability, impairs acylCer biosynthesis, and ultimately compromises barrier formation. This spatial disruption provides a mechanistic explanation for the generalized ichthyosis observed in affected individuals. Image was created in https://BioRender.com. ABHD5, alpha/beta-hydrolase domain-containing 5; acylCer, omega-*O*-acylceramide; DAG, diacylglycerol; ER, endoplasmic reticulum; LA, linoleic acid; mut, mutation; ω-OH-Cer, omega-hydroxy-ceramide; PLIN, perilipin; PNPLA1, patatin-like phospholipase domain-containing 1; TAG, triacylglycerol.

Under physiological conditions, PNPLA1 is thought to associate primarily with the endoplasmic reticulum (ER) membrane (11), from where it can interact with LDs to catalyze the transesterification of omega-hydroxy-ceramides with linoleic acid, generating acylCers (6). This reaction occurs in a microenvironment enriched in triacylglycerols, which supply the essential linoleic acid substrate. The correct spatial organization between PNPLA1 and LDs is therefore indispensable for efficient acylCer synthesis and, ultimately, for the formation of a competent epidermal barrier (Figure 1A).

Importantly, additional enzymes required for acylCer biosynthesis are likewise ER-associated. Fatty acid elongases ELOVL1 and ELOVL4 generate ultra-long-chain fatty acids (12, 13), which are then omega-hydroxylated by the cytochrome P450 enzyme CYP4F22 (14). Ceramide synthase 3 (CERS3) subsequently transfers these omega-hydroxy ultra-long-chain fatty acids onto sphingosine (15), producing omega-hydroxy-ceramides that serve as substrates for PNPLA1 in acylCer synthesis. Thus, acylCer formation emerges as a coordinated ER-centered process in which multiple sequential enzymatic steps occur within a shared subcellular compartment. In this setting, the spatial coupling of ER-associated reactions to LD-derived linoleic acid gains additional mechanistic significance.

A central regulator of this spatial coordination is ABHD5, a protein that was initially characterized as a co-activator for adipose triglyceride lipase (ATGL, also known as PNPLA2) in adipocytes (16). In keratinocytes, ABHD5 not only stimulates PNPLA1 catalytic activity but may also facilitate the recruitment or tethering of LDs to PNPLA1 through direct protein-protein interactions (7–9). This functional partnership ensures that PNPLA1 acts within the essential lipid microenvironment, bringing the enzyme into close proximity with its lipid substrates (Figure 1A).

The loss of ABHD5 function due to pathogenic mutations disrupts this essential spatial organization (9, 17). In the absence of ABHD5-mediated LD-PNPLA1 coupling, the enzyme loses access to its lipid substate pool (Figure 1B). The resulting defect in acylCer biosynthesis disrupts the assembly of the lipid lamellae in the *stratum corneum* (18) and, consequently, the formation of the epidermal barrier, which manifests clinically as a generalized ichthyosis marked by severe xerosis and hyperkeratotic scaling (19–23). Together, these findings highlight that the integrity of the epidermal barrier depends not only on PNPLA1 catalytic activity but equally on its correct spatial organization with LDs, a process orchestrated by ABHD5.

## ABHD5 as a determinant of subcellular proteostasis

At the mechanistic level, these observations demonstrate that ABHD5 fulfills a dual role: it not only co-activates lipid hydrolases and transacylases but also controls their access to lipid substrates. PNPLA1 by itself shows only minimal affinity for the limiting membrane of LDs, making ABHD5 essential for bringing LDs into functional proximity with the enzyme. Current evidence demonstrate that this spatial coordination relies on the ability of ABHD5 to associate with the hydrophobic LD surface – likely through perilipin binding – while also recognizing specific amino acid motifs within PNPLA1 to directly interact with the enzyme (9). Although the precise molecular architecture of this tethering mechanism is not yet fully understood, the available data suggest that ABHD5 functions less as a classical enzyme cofactor and more as a spatial organizer integrating LDs with ER-associated lipid synthesis.

A related targeting principle is well-established in adipocytes, where ABHD5 recruits ATGL to LDs to initiate lipolysis (16, 24, 25). In this setting, ABHD5 promotes lipid breakdown to meet cellular energy demands. In the epidermis, however, the same spatial logic appears redirected toward lipid synthesis rather than catabolic turnover. Here, ABHD5 facilitates the functional coupling of PNPLA1 to LDs, thereby enabling the synthesis of structural lipids essential for barrier integrity. This contrast suggests that ABHD5-mediated targeting represents a conserved organizational principle that is adapted in a tissue-specific manner to fulfill distinct metabolic functions. Together, these observations position ABHD5 as a central regulator of spatial enzyme coordination in mammalian lipid and energy metabolism.

## A paradigm shift—From enzymopathies to organelle targeting disorders

Inherited forms of autosomal recessive congenital ichthyosis have traditionally been regarded as disorders of lipid metabolism most often caused by defects in enzymes required for acylCer synthesis (26). Recent insights refine this concept. We propose a conceptual model in which ichthyosis resulting from mutations in *ABHD5* may represent a disorder of organelle targeting (Figure 1). In this model, ABHD5 itself remains functionally competent, yet its function becomes inaccessible due to a failure in spatial organization (9). Notably, forced relocalization of disease-associated ABHD5 variants together with PNPLA1 to the LD membrane restored functional acylCer synthesis (9), providing direct evidence that the defect arises from disrupted spatial organization rather than loss of intrinsic protein function. At present, this classification should be regarded as a mechanistic model that integrates available experimental evidence, while further studies across independent systems will be required to substantiate and generalize the concept.

From this perspective, the distinction between impaired protein function and spatial mislocalization has both diagnostic and conceptual significance. It suggests that the clinical manifestation of a genetic defect is determined not only by the intrinsic function of the affected protein, but equally by its subcellular localization. Two different mutations—one that impairs protein function and another that disrupts its spatial distribution—may therefore converge on the same metabolic endpoint, resulting in indistinguishable clinical phenotypes.

Within this conceptual model, ABHD5-syndromic epidermal differentiation disorder (ABHD5-sEDD, also known as Chanarin-Dorfman syndrome) can be classified among a broader group of “organelle diseases,” in which the mislocalization or mistargeting of otherwise functional proteins disrupts inter-organelle communication and metabolic control. Within this broader biological context, dermatologic disease shares pathogenic mechanisms with neurodegeneration and metabolic disorders, where defects in protein trafficking and compartmentalization emerge as primary drivers of pathology (27, 28).

## Implications for diagnosis and therapy

Recognizing ABHD5-sEDD as an organelle-targeting disorder calls for a reevaluation of current diagnostic approaches. In patients with congenital ichthyosis who lack mutations in established disease-associated genes, greater attention should be directed toward factors that regulate subcellular trafficking and organelle dynamics. In the case of ABHD5-sEDD, immunolocalization studies of ABHD5 and PNPLA1 in keratinocyte cultures or patient-derived skin biopsies may provide valuable diagnostic insight complementary to conventional genomic analysis.

From a therapeutic perspective, restoring the correct spatial organization of the ABHD5-PNPLA1 complex provides a potential strategy to recover enzymatic function, even in the presence of otherwise intact catalytic activity. Pharmacological approaches that stabilize the ABHD5-PNPLA1 interaction or promote proper organelle coupling, may help to restore functional acylCer synthesis. In addition, small molecules that modulate LD surface properties to enhance PNPLA1-LD recruitment could further support this process. Complementary to these mechanistic approaches, topical delivery of synthetic acylCer analogs might compensate for the underlying biosynthetic defect in ABHD5-sEDD and partially restore epidermal barrier integrity.

## Lipid droplet dynamics and spatial organization in epidermal differentiation

The epidermis provides an elegant example of how metabolic specialization is closely linked to cellular differentiation. During terminal differentiation of basal keratinocytes into corneocytes, LDs undergo profound remodeling: their number increases, their proteome changes, and they eventually coordinate with other cellular compartments involved in epidermal lipid metabolism (29–31). Far from acting as passive lipid stores, LDs function as dynamic metabolic platforms that integrate intracellular lipid storage with the assembly of the extracellular barrier.

Central to this process, ABHD5 orchestrates the access of PNPLA1 to its lipid substrates, ensuring efficient synthesis of acylCers required for skin barrier integrity (9). When ABHD5 is absent or dysfunctional, the spatial organization between LDs and the ER is disrupted, impairing the acylCer production and ultimately compromising barrier formation.

This example underscores a broader principle in lipid biology, which highlights that the localization of enzymes and other lipid-metabolizing proteins is as crucial as their intrinsic activity. The ABHD5-PNPLA1 axis exemplifies this concept by showing that the mislocalization of an otherwise functional protein can mimic the biochemical and clinical features of a congenital lipid deficiency. Recognizing organelle targeting as a primary pathogenic mechanism not only expands diagnostic and therapeutic approaches for skin disease but also suggests that similar spatial principles may control lipid metabolism in other epithelial tissues. Understanding how LD dynamics coordinate with keratinocyte differentiation provides a basis for interventions aimed at restoring both molecular function and the spatial organization essential for barrier integrity.

## Conclusion

The emerging view that intracellular organization defines metabolic function invites a broader reevaluation of how skin barrier formation is regulated. The link between LDs and the ER suggests that the epidermis relies on a finely tuned spatial sequence of events rather than on isolated enzymatic reactions. This mechanistic model places keratinocyte differentiation within a network of coordinated organelle interactions that collectively maintain barrier integrity.

Beyond the epidermis, such spatial principles may apply to other epithelia where lipid synthesis, modification, and secretion are tightly linked with cellular differentiation. Exploring these parallels could reveal shared regulatory mechanisms and identify additional factors involved in organelle communication and lipid transport.

A next key step will be to translate these mechanistic insights into diagnostic and therapeutic strategies. For example, assessing the subcellular distribution of key lipid-metabolizing enzymes in patient-derived material could provide diagnostic evidence that complements genomic analyses. Likewise, approaches that modulate the physicochemical properties of LDs or stabilize interactions between LD-associated proteins may open new strategies for therapy.

More generally, recognizing that the mislocalization of functional proteins can itself be pathogenic extends the concept of disease beyond the classical paradigm of enzymopathies. It links dermatologic disorders to a broader biological principle according to which the loss of spatial organization, whether in adipocytes, neurons, or keratinocytes, can compromise tissue function. This broader view may guide future research into how cellular organization contributes to maintaining and restoring skin integrity.
